# Evaluation of the effectiveness of four alternative approaches for the classical cough test during a urodynamic study in the context of the COVID-19 global pandemic

**DOI:** 10.1186/s12894-023-01296-5

**Published:** 2023-07-25

**Authors:** Xiao Zeng, Shuang Liu, SiHong Shen, Hong Shen, De-yi Luo

**Affiliations:** 1 Department of Urology, Institute of Urology, West China Hospital, Sichuan University, Chengdu, China; 2 West China School of Nursing, Sichuan University, Chengdu, China

**Keywords:** Urodynamic study, COVID-19, Cough test, Alternative approach, International Continence Society, Face mask

## Abstract

**Backgroud:**

To evaluate four different alternatives to the classical cough test during a urodynamic study in the context of the COVID-19 pandemic.

**Methods:**

Patients who needed to undergo a urodynamic study (UDS) at the West China Hospital of Sichuan University between April 2021 and May 2021 were randomly selected according to the inclusion and exclusion criteria. During the UDS process, we used four alternative methods to the “cough test”: 1) quickly pressing the bladder area, 2) performing the Valsalva maneuver, 3) performing the Kegel maneuver, and 4) letting the patient close their mouth while performing the cough test. The "cough" waveform amplitudes and characteristics of the graphics were obtained and compared with the classical cough test.

**Results:**

A total of 120 patients (89 men, 31 women) were included in the study. There was no significant difference between the cough waveform amplitude induced by the Valsalva maneuver compared with the classical cough test (*P* = 0.182); there was no significant difference between the cough waveform amplitude induced by the cough test with the mouth closed and the classical cough test (*P* = 0.342); there was no significant difference between pressing quickly on the bladder area and the classical method (*P* = 0.076); and there was a significant difference between the data obtained by the Kegel maneuver and the classical method (*P* < 0.05). The average "cough" amplitudes obtained were 73.14 ± 22.48 cm H2O, 66.17 ± 17.12 cm H2O, 82.93 ± 18.95 cm H2O, 26.50 ± 8.68 cm H2O, and 68.90 ± 20.32 cm H2O by the classical cough test, by quickly pressing the bladder area, by the Valsalva maneuver, by the Kegel maneuver, and by coughing with a closed mouth, respectively.

**Conclusion:**

Quickly pressing the bladder area, performing the Valsalva maneuver, and letting the patient close their mouth while performing the cough test can all provide effective cough waveforms and amplitudes.

**Trial registration:**

No. 2021–183.

## Background

Urodynamic studies (UDS) are generally used to describe measurements that assess the function and dysfunction of the lower urinary tract using an appropriate method [1]. In 2002, the International Continence Society (ICS) published the first version of the “Good Urodynamic Practices (GUP) [2],” with updated versions published in 2013–2015 and 2015–2016 [3]. The GUP is the official international guidelines for UDS, which is currently the most recognized urodynamic technical guidelines in the world [2]. In the “Pressure Signal Quality Control: Qualitative and Quantitative Plausibility” section of the GUP, the minimum criteria for urodynamic quality control are specified. This section clarifies that the “cough” test was an important way to check whether the signal is “alive.” The GUP also emphasize that we must perform cough tests throughout the entire UDS process; from the beginning to the end of the test, cough tests should be performed once for every 50 mL of filled volume.

However, in December 2019, a highly contagious viral pneumonia caused by the novel coronavirus disease 2019 (COVID-19) was first reported in Wuhan, China [4], which soon spread throughout China; subsequently, COVID-19 cases were reported around the world, and the World Health Organization (WHO) declared the outbreak of a public health emergency [5]. Generally, infected people spread virus particles when they speak, breathe, cough, or sneeze [6]; similar to other viral diseases, the main transmission routes include person-to-person transmission, airborne transmission, and other modes of transmission [7]. The airborne transmission potential of the COVID-19 virus must be considered in prevention and control efforts, and the transmission of viral aerosols must be effectively reduced by wearing protective equipment [8]. Because the COVID-19 virus is highly contagious, being in contact with infected patients or carriers of the virus becomes very dangerous, exposing health providers to a high risk of infection.

To ensure the quality of UDS, we must conduct multiple cough tests during the entire UDS process to verify whether the signal is “alive”.A patient who is repeatedly coughing inadvertently increases the chance of virus transmission even with some basic protection measures. Reducing the risk of exposure of urodynamicists during UDS is very important. In addition, by following the standard protection approach, such as wearing masks and protective face screens, this problem can also be solved by finding alternative approaches to replace the classical cough test. The purpose of this study was to evaluate the effectiveness of some alternatives that may replace the classical cough test for COVID-19.

## Methods

Patients who needed to undergo UDS at the West China Hospital of Sichuan University between April 2021 and May 2021 were randomly selected according to the inclusion criteria.

### Inclusion criteria

Patients who were selected for the study met the following study enrollment criteria: 1) aged > 18 years; 2) had a clear medical history; 3) had normal cognitive function and could cooperate with researchers; 4) had no surgical history of the anus, abdomen, lungs, throat, or pelvic floor area; 5) had negative nucleic acid test results for COVID-19; and 6) had a clear urodynamic trace.

### Exclusion criteria

Patients who met the following criteria were excluded from the study: 1) patients who could not cooperate with the researcher's instructions, 2) patients who had been previously diagnosed with COVID-19, and 3) patients who were diagnosed with other respiratory infections.

### Urodynamic study process

All UDS were performed using catheters with standard measurements and UDS equipment with an air charged system (ACS); sterile saline (37 °C) was used as the filling medium. For patients with nonneurogenic bladder dysfunction, the filling rate was 60–90 mL/min; for patients with neurogenic bladder dysfunction, the filling rate was 10–30 mL/min. All patients were in a sitting position when undergoing the test, and all UDS procedures strictly referred to the GUP guidelines [2, 9]. During the UDS process, the "cough" waveform data were obtained using five “cough test” methods: 1) classical cough tests, 2) quickly pressing the bladder area, 3) performing the Valsalva maneuver, 4) performing the Kegel maneuver, and 5) letting the patient close their mouth while performing the cough test.

### Medical ethics approval

This study was approved by the Medical Ethics Committee of the West China Hospital of Sichuan University (2021–183), and the study was conducted in strict accordance with the Declaration of Helsinki. All enrolled patients signed an informed consent form.

### Statistical analysis

Statistical analysis was performed using SPSS software. The “cough” wave amplitudes from the five different approaches are presented as the mean ± SD. The Komogorov-Smirnov approach was used to test whether the data obeyed a normal distribution. A paired t test was used for the statistical comparison of urodynamic results. Statistical significance was set at *P* < 0.05.

## Results

### Baseline information of enrolled patients

A total of 120 patients (89 men, 31 women) were included in the study, with an average age of 42 ± 22.45 years and 51 ± 14.58 years for male and female patients, respectively. Among the patients, 22 were diagnosed with neurogenic bladder dysfunction, and 98 were diagnosed with nonneurogenic bladder dysfunction (Table 1).

**Table 1:** Baseline data of enrolled patients

Gender	Case Number(*n*)	Age(mean ± SD^a^)	Neurogenic bladder(*n*)	None-neurogenic bladder(*n*)	Air catheter system
Male	89	42 ± 22.45	13	76	89
Female	31	51 ± 14.58	9	22	31

^*a*^*SD* Standard Deviation

### Characteristics of “cough” waveform amplitudes obtained by the five different cough methods

The five different cough methods described in Urodynamic Study Process Section were performed during the UDS of all 120 enrolled patients, and the different amplitudes were recorded. The average "cough" amplitudes obtained were 73.14 ± 22.48 cm H2O, 66.17 ± 17.12 cm H2O, 82.93 ± 18.95 cm H2O, 26.50 ± 8.68 cm H2O, and 68.90 ± 20.32 cm H2O by the “Classical cough test”, by “Quickly pressing the bladder area”, by “Performing the Valsalva maneuver”, by “Performing the Kegel maneuver”, and by “Letting the patient close their mouth while performing the cough test”, respectively. We compared the cough amplitude obtained by the classical cough test with those obtained by the other cough test methods. According to the Komogorov-Smirnov test, all the data obeyed a normal distribution. Further analysis showed that there was no significant difference between the data obtained by the “classical cough test” and by “quickly pressing the bladder area” (*P* = 0.076), by “performing the Valsalva maneuver” (*P* = 0.182), or by “letting the patient close their mouth while performing the cough test” (*P* = 0.342). However, there was a significant difference between the data obtained by “performing the Kegel maneuver” and the “classical cough test” (*P* < 0.05) (Table 2).

**Table 2:** Different kinds of alternatives compared with the classical cough test

Approach	Amplitude(mean ± SD^b^cmH20)	*P* value	Case Number(*n*)
Cough test(classic)	73.14 ± 22.48	N/A^a^	120
Quickly pressing the bladder area	66.17 ± 17.12	0.076	120
Performing the Valsalva maneuver	82.93 ± 18.95	0.182	120
Performing the Kegel maneuver	26.50 ± 8.68	< 0.05	120
Letting the patient close their mouth while performing a cough test	68.90 ± 20.32	0.342	120

ALL urodynamic study system was air catheter system (ACS); *P* < 0.05 shows statistically significant; 1cmH20 = 0.133kpa

^a^*N/A* Not Available

^b^*SD*: Standard Deviation

### The “cough” waveform data obtained by the five different cough test methods compared with the initial intravesical pressure

The data obtained by the “classical cough test”, by “quickly pressing the bladder area”, by “performing the Valsalva maneuver”, and by “letting the patient close their mouth while performing the cough test” were significantly different from that of the initial intravesical pressure (*P* < 0.05); however, there was no significant difference between the data obtained by “performing the Kegel maneuver” and the initial intravesical pressure (*P* = 0.587) (Table 3).

**Table 3:** The “cough” waveform data obtained by the five different cough methods compared with the initial intravesical pressure

Methods	Amplitude (mean ± SD^#^cmH20)	*p*
Initial intravesical pressure (mean ± SD^a^cmH20)	25.5 ± 5.07	NA^*^
Cough test(classic)	75.13 ± 22.38	< 0.05
Quickly pressing the bladder area	66.17 ± 17.12	< 0.05
Performing the Valsalva maneuver	82.93 ± 18.95	< 0.05
Performing the Kegel maneuver	26.50 ± 8.68	0.587
Letting the patient close their mouth while performing a cough test	68.90 ± 20.32	< 0.05

^a^*SD* Standard Deviation

NA* Not available.1cmH20 = 0.133kpa

*P* < 0.05 shows statistically significant

### Morphological characteristics of different subtypes of “cough” waveforms

In this study, we found that several “cough waveforms” induced by “alternative approaches” often represent four different subtypes. In particular, we found that the waveform induced by “performing the Valsalva maneuver” often appeared in two types: the Type 3 waveform, characterized by a “towering shape” and “sharp shape”, and the Type 4 waveform, characterized by a “platform shape.” Regarding the Type 3 waveform induced by the “Valsalva maneuver”, this type of waveform is expected to be tall and pointed, resulting in a "towering shape" and "sharp shape"; this kind of fluctuation is often caused by rapid Valsalva movement. The Valsalva maneuver can also induce a different waveform, which is characterized by a “platform shape”, called the Type 4 waveform, when compared with the Type 3 waveform. The main feature of the Type 4 waveform is that the waveform is trapezoidal and has a long plateau pattern change, and these graphical features are often due to a longer period of Valsalva movement. In real-world clinical practice, the two different types of waveforms described above may occur due to the individual differences of patients.

**Fig. 1:**
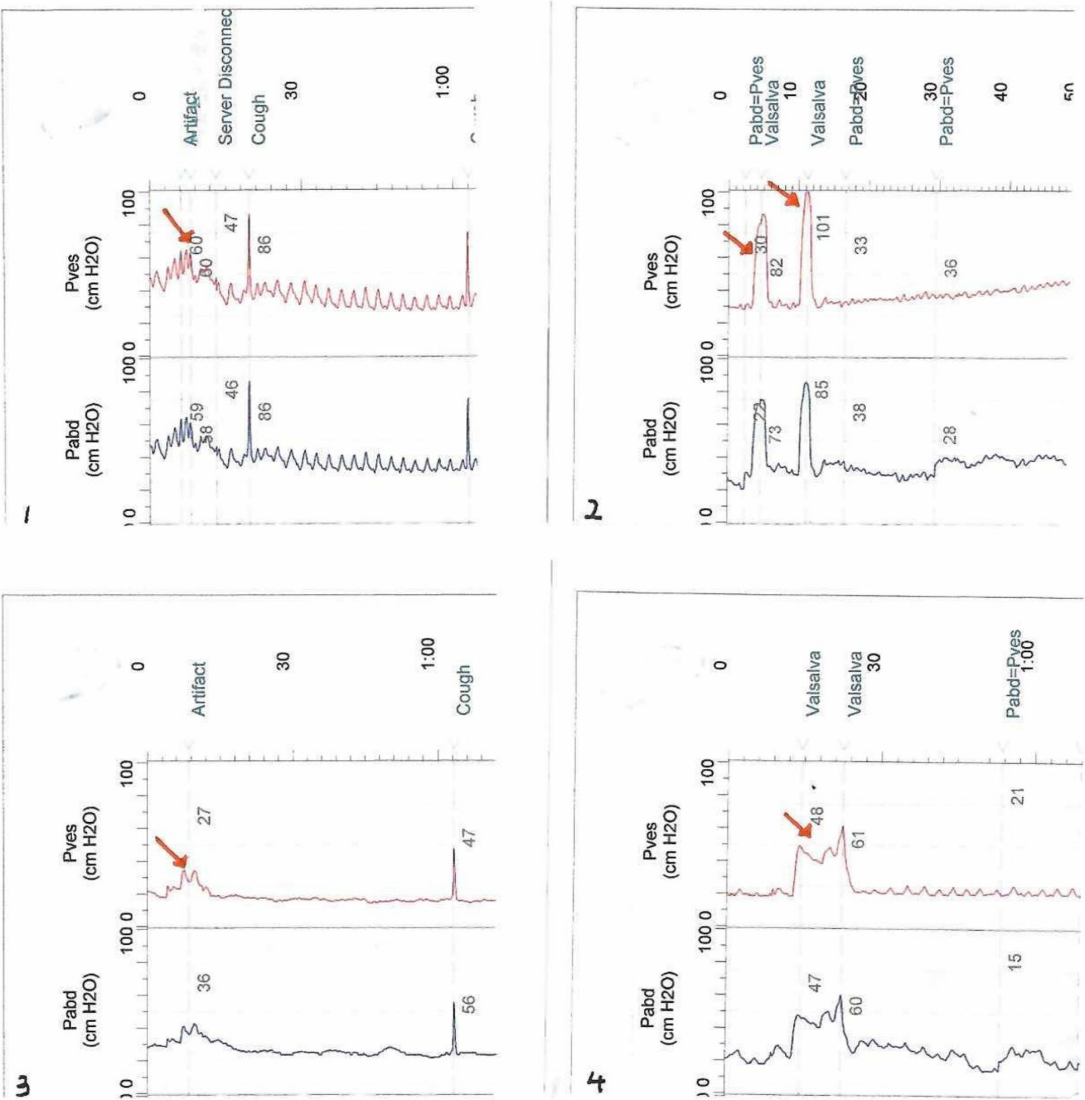
Characteristics of different types of “cough” waveforms

We also found two subtypes in the waveforms induced by “performing the Kegel maneuver”: the Type 1 waveform, characterized by a “low waveform shape”, and the Type 2 waveform, characterized by a “weak waveform shape” (Fig. 1). When compared with these waveforms obtained by the “Valsalva maneuver”, the general characteristic of the waveforms induced by “performing the Kegel maneuver” are that their amplitudes are low, and some amplitudes can even be difficult to observe and record. This may be related to the Kegel maneuver, which mainly stimulates the pelvic floor muscles rather than directly increasing the abdominal pressure; therefore, the waveform amplitude is significantly lower than that obtained by directly increasing the abdominal pressure, such as when performing the “Valsalva maneuver” mentioned above.

### The morphological characteristics of the “cough” waveforms obtained by the five different methods in the same UDS process

**Fig. 2:**
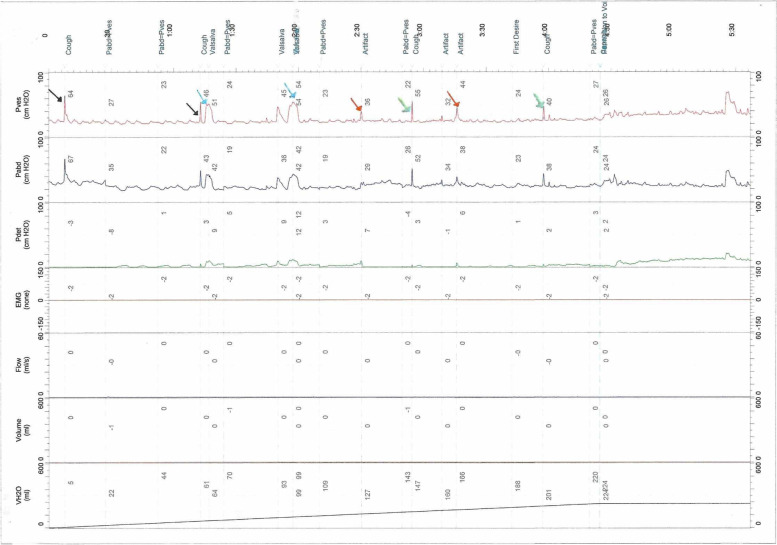
The "cough wave pattern" generated by different alternative approaches in the same UDS process

All five methods could produce “cough” waveforms, but when compared with the waveform amplitude induced by “classical cough tests”, the waveform amplitude induced by “performing the Kegel maneuver” was generally smaller and sometimes more difficult to observe and record. “Performing the Valsalva maneuver” method will always produce a higher waveform amplitude than other methods. Compared to the “sharp shape” waveform obtained by the “classical cough test”, the waveform obtained by “performing the Valsalva maneuver” had a “high platform shape”. Waveforms obtained by “letting the patient close their mouth while performing the cough test” and by “quickly pressing the bladder area” were similar to those obtained by the “classical cough test” (Fig. 2).

## Discussion

The rapid spread of COVID-19 is attributed to airborne particles exhaled by infected but often asymptomatic individuals [10]. Personal protective equipment (PPE) is commonly used to filter airborne pathogenic particles that can be inhaled into the lungs. In the era of COVID-19, the role of face masks has expanded to reduce aerosol emissions from the respiratory system because airborne particles have been identified as one of the main routes of infection [11, 12]. Although PPE can be the primary approach for preventing the spread of COVID-19 between doctors and patients, it may be a better choice to reduce the transmission rate by reducing the formation of iatrogenic aerosols, such as by substituting the classical cough test with some effective alternative methods. Only by fundamentally reducing the generation of potential pathogenic aerosols can urodynamicists be kept safe. This study mainly verified four alternatives to the classical cough test for quality control.

The Valsalva test plays an important role in the UDS of stress urinary incontinence (SUI). In 1993, McGuire's study showed that the Valsalva Leak Point Pressure could be used to identify the different types of SUI [13]. The main mechanism of the Valsalva test is to induce the appearance of SUI by increasing the abdominal pressure, and during the Valsalva maneuver, increased intra-abdominal pressure is generated and expelled toward the urethra [14]. However, the past use of the Valsalva maneuver in the urodynamic field has been in the auxiliary diagnosis of stress urinary incontinence rather than in the quality control of urodynamic studies. Because the Valsalva maneuver can directly increase patients’ abdominal pressure, this is consistent with the principle of waveforms induced by increasing abdominal pressure through the cough test in our current urodynamic quality control process. This common ground has also led researchers to study these two methods. A study showed that if a poor quality cough occurs and is not resolved by corrective measures, the Valsalva maneuver is recommended to further assess whether transmission and hence, urodynamic quality is satisfactory. This study also verified the effectiveness of the Valsalva maneuver method [15]. In the present study, we found that there was no significant difference between the cough waveform induced by “performing the Valsalva maneuver” compared with that of the classical cough pattern (*P* = 0.182); however, as shown in the data, the average amplitude of the “cough pattern” induced by “performing the Valsalva maneuver” was higher than that of the “classical cough test” (82.93 ± 18.95 vs. 73.14 ± 22.48), which was also the highest among the four alternative methods. The cough waveforms induced by “performing the Valsalva maneuver” had a “platform shape”. “Quickly pressing the bladder area” has a similar mechanism as “performing the Valsalva maneuver”, which can also obtain a cough waveform similar to that of the classical cough method (66.17 ± 17.12 vs. 73.14 ± 22.48). This method is also commonly used in children who are unable to comply with the classical cough test. Coughing with a closed mouth can obtain a waveform that is mostly similar to that of the classical cough test (68.90 ± 20.32 vs. 73.14 ± 22.48), but this approach has the same limitations as the classical cough test; for example, some children or deaf-mute patients cannot cooperate with urodynamicists’ instructions. Because in the process of performing these alternative approaches, patients do not need to open their mouths, both approaches can reduce the production of aerosols that may contain potential pathogens.

Pelvic floor muscle exercises have been the first-line treatment for pelvic floor dysfunction since Arnold Kegel introduced them half a century ago [16]. Their main principle is to increase the function of the pelvic floor muscles through training. The adult urinary bladder is located anteriorly in the pelvic cavity and behind the pubic bones [17]. Therefore, the Kegel maneuver cannot directly increase abdominal pressure; it can only indirectly increase abdominal pressure through pelvic floor muscle contraction, which may lead to a decrease in the abdominal pressure signal. In this study, we found that although “performing the Kegel maneuver” could induce the waveform, the amplitude of the obtained waveforms was small (26.50 ± 8.68 vs. 73.14 ± 22.48), and some of them could not even be identified. The difference was statistically significant (*P* < 0.05) when compared to the classical cough test. However, when compared with the initial intravesical pressure, there was no statistically significant difference, which means that the alternative approach was not sufficient to obtain the waveform signal. Concurrently, the pelvic floor muscles are in the pelvic cavity, and patients may intuitively feel them; thus, many patients cannot accurately contract their pelvic floor muscles. Clinicians usually use biofeedback therapy to help patients feel their pelvic floor muscles [18]. There is a significant difference between the Kegel method and the classical method; because most patients cannot accurately complete the Kegel maneuver, we do not think it is an effective alternative method.

We preliminarily verified the effectiveness of “Quickly pressing the bladder area”, “Performing the Valsalva maneuver”, and “Letting the patient close their mouth while performing the cough test”. However, due to the great individual differences of patients in real-world clinical practice, we can choose suitable alternative methods according to the different actual conditions of patients. For example, for patients with good cooperation, we recommend the Valsalva maneuver instead of the classical cough test to obtain a waveform. For some patients who do not cooperate well, including children, patients with cognitive impairment, patients with Alzheimer's disease, etc., “Quickly pressing the bladder area” may be a better choice to obtain a waveform. For patients with poor cooperation, it is also not suitable to choose the “Letting the patient close their mouth while performing the cough test” approach. Appropriate alternative methods should be chosen according to the specific situation of a patient.

It should be emphasized that regardless of which of the above alternatives are used, PPE remains the most basic and effective form of infection prevention. In our single-center experience, we used surgical or N95 masks and protective face screens as the standard prevention approach. We also routinely request patients take a COVID-19 nucleic acid test before undergoing the UDS, although we are aware that due to the cost of nucleic acid testing, it cannot be carried out in all areas. The findings of this study aim to have a positive effect on reducing urodynamicists' risk of COVID-19 infection. These alternative methods can reduce the production of aerosols that may contain potentially pathogenic microorganisms, which can effectively reduce the direct transmission of the virus between medical staff and patients.

## Conclusions

Through this study, we preliminarily verified that “Quickly pressing the bladder area”, “Performing the Valsalva maneuver”, and “Letting the patient close their mouth while performing the cough test” can obtain effective cough waveforms and amplitudes. These can all be alternative approaches to the classical cough test in the context of the COVID-19 pandemic.

## Data Availability

The datasets used and/or analysed during the current study are available from the corresponding author on reasonable request.

## Abbreviations

UDS: Urodynamic study
ICS: International continence society
GUP: Good urodynamic practices
COVID-19: Novel coronavirus disease 2019
WHO: World health organization
ACS: Air charged system
PPE: Personal protective equipment
SUI: Stress urinary incontinence
